# Prescribing preferences in rapid tranquillisation: a survey in Belgian psychiatrists and emergency physicians

**DOI:** 10.1186/s13104-015-1172-2

**Published:** 2015-06-05

**Authors:** Chris Bervoets, Ella Roelant, Jürgen De Fruyt, Hella Demunter, Barry Dekeyser, Leen Vandenbussche, Koen Titeca, Guido Pieters, Bernard Sabbe, Manuel Morrens

**Affiliations:** Faculteit geneeskunde en gezondheidswetenschappen, Collaborative Antwerp Psychiatric Research Institute (CAPRI), University of Antwerp, Antwerp, Belgium; UPC KULeuven, Campus Gasthuisberg, Louvain, Belgium; StatUa Center for Statistics, University of Antwerp, Antwerp, Belgium; AZ Sint Jan Brugge-Oostende AV, Brugge, Belgium; UPC KULeuven, Campus Kortenberg, Louvain, Belgium; AZ Herentals, Herentals, Belgium; UZ Gent, Ghent, Belgium; AZ Groeninge, Kortrijk, Belgium; PZ Sint Norbertus, Duffel, Belgium; PZ Boechout, Boechout, Belgium

**Keywords:** Agitation, Rapid tranquillisation, Emergency psychiatry, Guidelines

## Abstract

**Background:**

The pharmacotherapeutic management of agitation is a common clinical challenge. Pharmacotherapy is frequently used, the use of published guidelines is not known. The purpose of this study was twofold; to describe the prescribing patterns of psychiatrists and emergency physicians and to evaluate to which extent guidelines are used.

**Methods:**

A cross-sectional survey in the Dutch-speaking part of Belgium is carried out in 39 psychiatric hospitals, 11 psychiatric wards of a general hospital and 61 emergency departments. All physicians are asked for demographic information, their prescribing preferences, their use of guidelines and the type of monitoring (effectiveness, safety). For the basic demographic data and prescription preferences descriptive statistics are given. For comparing prescribing preferences of the drug between groups Chi square tests (or in case of low numbers Fisher’s exact test) were performed. Mc Nemar test for binomial proportions for matched-pair data was performed to see if the prescription preferences of the participants differ between secluded and non-secluded patients.

**Results:**

550 psychiatrist and emergency physicians were invited. The overall response rate was 20% (n = 108). The number 1 preferred medication classes were antipsychotics (59.3%) and benzodiazepines (40.7%). In non-secluded patients, olanzapine (22.2%), lorazepam (21.3%) and clotiapine (19.4%) were most frequently picked as number 1 choice drug. In secluded patients, clotiapine (21.3%), olanzapine (21.3%) and droperidol (14.8%) were the three most frequently chosen number 1 preferred drugs. Between-group comparisons show that emergency physicians prefer benzodiazepines significantly more than psychiatrists do. Zuclopenthixol and olanzapine show a particular profile in both groups of physicians. Polypharmacy is more frequently used in secluded patients. Published guidelines and safety or outcome monitoring are rarely used.

**Conclusions:**

Our results show that prescription practice in Flanders (Belgium) in acute agitation shows a complex relationship with published guidelines. Prescription preferences differ accordingly to medical specialty. These findings should be taken into account in future research.

## Background

Agitated behaviour during a hospital admission is a frequently encountered problem. It is estimated that yearly 1.7 million hospital admissions occur due to agitation [1, 2]. About 10% of the patients admitted to the emergency department are at risk of developing agitation symptoms [3]. Agitation is seen in conjunction with different psychiatric and non-psychiatric medical conditions [4–6].

Although motor restlessness, increased responsiveness to stimuli, irritability, inappropriate and usually purposeless verbal and motor activity are reported as the major hallmarks of the syndrome, there is no consensus regarding the symptoms that constitute agitation. Little is known about its course over time and which level of agitation should lead to pharmacological treatment [7, 8]. This ambiguities all hamper the development of generalizable clinical guidelines [9]. Although good clinical trials have been conducted to investigate the effectiveness and the safety of drugs prescribed in agitated behaviour [10–12], results should be interpreted cautiously in clinical practice, given that these trials have better internal than ecological validity [13–15]. Consequently, prescribing preferences may not always be based on evidence based guidelines, although the use of drugs in this indication is widely spread [1, 2, 9, 15].

In order to map prescribing habits of physicians in acute agitation, a series of studies—surveying physicians’ preferences—was conducted between 1999 and 2004 [10, 12, 16–20]. While they all differ in methodological design, do not compare between psychiatrists and emergency physicians, and are all service-specific or region specific, a few trends can be found in these reports. Firstly, antipsychotics (olanzapine, haloperidol and droperidol) and benzodiazepines (diazepam, lorazepam) are the preferred drugs of choice. Secondly, there is no real preference for peroral or intramuscular administration. Thirdly, there is a reported trend in favour of using combinations of drugs.

The present study was conducted with a twofold objective. We firstly aimed to describe the prescribing preferences and their between group differences of psychiatrists and emergency physicians in Belgium in the management of acute agitation and secondly we evaluated to which extend prescribing preferences were in accordance with published treatment guidelines.

## Methods

A cross-sectional online survey was carried between the July 2012 and September 2012 in 39 psychiatric hospitals, 11 psychiatric wards of a general hospital and 61 emergency departments. 281 psychiatrists and 267 emergency physicians received an invitation to respond to the survey. Participating psychiatrists and emergency physicians were asked to give demographic and professional information, describe their prescribing habits in the treatment of acute agitation, their use of evidence based guidelines and the type of monitoring (effectiveness, safety) they provide for their patients. Agitation was defined in the survey as a clinical condition with acute onset of psychomotor and emotional excitement. The following parameters were collected: age, gender, medical specialty, medical setting, number of patients with agitation admitted in 1 month, use of rating scales, preference of drug prescribed, use of drugs in combinations, effect of seclusion on prescription preference, use of guidelines and modalities of monitoring of efficacy as well as patient safety.

First, it was evaluated which medication classes and specific drugs were preferred and if preferences changed in the case the patient needed seclusion. Second, we looked for differences in prescribing habits depending on the medical specialty.

For the basic demographic data and prescription preferences descriptive statistics are given. For each drug, two Chi square tests (or in case of low numbers Fisher’s exact test) were performed, one comparing prescribing preferences of the drug between psychiatrists and emergency physicians and another one comparing prescribing preferences between age groups. Mc Nemar test for binomial proportions for matched-pair data was performed to see if the prescription preferences of the participants differ between secluded and non-secluded patients. IBM SPSS Statistics version 20.0 was used for statistical analyses.

## Results

### Response rate and respondents characteristics

110 psychiatrists and emergency physicians responded to the online survey, yielding a response rate of 20%. Two of them were removed from further analysis as they reported treating 0 patients per month for agitation. From the 108 respondents, 69 (63.9%) were male and 39 (36.1%) were female. Psychiatrist accounted for 65.7%, emergency physicians for 34.3%. Sixty-seven respondents were between the ages of 25 and 45 years of age, 41 respondents were older than 45 years. Physicians worked in different settings; 42 (38.9%) in a psychiatric hospital, 25 (23.1%) in a psychiatric ward of a general hospital and 41 (38.0%) in an emergency service. A caseload for agitation between 1 and 10 patients per month was reported by 69 (63.9%) of the participants, 23 (21.3%) had a monthly caseload between 11 and 20 and 16 (14.8%) reported a caseload higher than 20 patients.

### Preferences in medication prescriptions

#### General preferences for a medication class in acute agitation

Respondents were asked to point out which of five medication classes (antipsychotics, antidepressants, benzodiazepines, mood stabilizers and antihistaminics) they favoured in the treatment of acute agitation. They had to give the classes a number from 1 to 5 with 1 being the most preferred class, 2 the second preferred class,… All respondents chose either antipsychotics (59.3%) or benzodiazepines (40.7%) as their number 1 preferred medication. As a number 2 medication class benzodiazepines were most preferred (54.6%). The number 3 medication class that was most popular are the mood stabilizers (37.4%), number 4 are the antidepressants (38.3%) and number 5 are the antihistaminics (44.9%).

Rankings for all medication classes are shown in Table 1.

**Table 1:** Overview of number 1–5 preferred medication classes

AP	BZD	AD	MS	AH
59.3	40.7	0	0	0
40.7	54.6	1.9	1.9	0.9
0	1.9	31.8	37.4	29.0
0	1.9	38.3	34.6	25.2
0	0.9	28.0	26.2	44.9

*AP* antipsychotics, *BZD* benzodiazepines, *AD* antidepressants, *MS* mood stabilizers, *AH* antihistaminics.

#### Prescribing preferences in non-secluded patients

All participants were given a list of 80 drugs and were asked to select their 3 most used drugs when considering all patients in need for a treatment of agitation and rank these according to preference, rank 1 being their first choice drug. All rankings are listed in Table 2. Olanzapine (22.2%), lorazepam (21.3%) and clotiapine (19.4%) were the three most popular first choice drugs. Most frequent ranked second were lorazepam (21.3%), olanzapine (17.6%) and droperidol (12.0%). As a third choice, clotiapine (13%), lorazepam (11.1%) and olanzapine (11.1%) were reported most frequently. Although zuclopenthixol does not appear but in third choice (2.8%), the long acting formula of this drug is reported more frequently (1.9% as first choice; 2.8% as second choice; 8.3% as third choice).

**Table 2:** Prescribing preferences in non-secluded and secluded patients

Drug	Non-secluded patients						Secluded patients					
	Rank 1		Rank 2		Rank 3		Rank 1		Rank 2		Rank 3	
	Frequency	%	Frequency	%	Frequency	%	Frequency	%	frequency	%	Frequency	%
Alprazolam	2	1.9			5	4.6	1	0.9	1	0.9	4	3.7
Alprazolam (LA)					1	0.9						
Amisulpiride											1	0.9
Aripiprazole	3	2.8	1	0.9	3	2.8	4	3.7			1	0.9
Bromazepam			1	0.9							1	0.9
Clonazepam					1	0.9					2	1.9
Clorazepaat	4	3.7	5	4.6	7	6.5	4	3.7	6	5.6	7	6.5
Clotiapine	21	19.4	8	7.4	14	13	23	21.3	13	12.0	11	10.2
Cloxazolam									1	0.9		
Diazepam	5	4.6	10	9.3	5	4.6	6	5.6	15	13.9	5	4.6
Droperidol	7	6.5	13	12	8	7.4	16	14.8	12	11.1	11	10.2
Escitalopram			1	0.9	1	0.9						
Haloperidol	7	6.5	3	2.8	10	9.3	4	3.7	7	6.5	13	12.0
Lamotrigine											1	0.9
Lorazepam	23	21.3	23	21.3	12	11.1	14	13	24	22.2	14	13
Midazolam	2	1.9	2	1.9	2	1.9	2	1.9	3	2.8	2	1.9
Olanzapine	24	22.2	19	17.6	12	11.1	23	21.3	12	11.1	8	7.4
Paliperidone					1	0.9					1	0.9
Pipamperone			3	2.8	1	0.9			2	1.9		
Prazepam					1	0.9					1	0.9
Promethazin			1	0.9					1	0.9	3	2.8
Quetiapine	5	4.6	12	11.1	5	4.6	3	2.8	6	5.6	3	2.8
Risperidone	2	1.9	2	1.9	3	2.8			1	0.9	2	1.9
Trazodone			1	0.9								
Sodium valproate	1	0.9			4	3.7	1	0.9			2	1.9
zuclopenthixol					3	2.8					4	3.7
zuclopenthixol (LA)	2	1.9	3	2.8	9	8.3	7	6.5	4	3.7	11	10.2
Total	108	100	108	100	108	100	108	100	108	100	108	100

*LA* long acting.

Of the participants 107 answered the question on the use of monotherapy versus combinations, 21.5% of them reported to use only monotherapy whilst 67.3% use a combination of drugs in a step-up regimen and 11.2% a combination of drugs from the start of the treatment.

#### Prescribing preferences in secluded patients

We also investigated what the prescription preferences were when patients were considered that are in need of a seclusion room as a non-pharmacological approach to agitation. 62% of the participants adapt their drug choice (38% of participants reported not to change their prescription preferences in this type of patients). Participants were asked to give their top 3 drugs for secluded patients from the same list of 80 drugs (rankings are shown in Table 2). Clotiapine (21.3%), olanzapine (21.3%) and droperidol (14.8%) were reported the most frequently as first choice. Most frequently ranked second were lorazepam (22.2%), diazepam (13.9%) and clotiapine (12.0%). Most mentioned number 3 drugs were lorazepam (13.0%), haloperidol (12.0%), clotiapine (10.2%), droperidol (10.2%) and zuclopenthixol long formula (10.2%). Again, zuclopenthixol does not appear but as third choice (3.7%). However, the long acting formula of this drug is reported more frequently than in the non-secluded group of patients (6.5; 3.7; 10.2%).

For the treatment of secluded patients 107 respondents answered the question on the use of monotherapy versus combinations. 15.9% reported to use only monotherapy whilst 59.8% use a combination of drugs in a step-up regimen and 24.3% a combination of drugs from the start of the treatment.

#### Differences in medication preferences for psychiatrists and emergency physicians

For non-secluded patients all participants ranked either antipsychotics or benzodiazepines as their first choice used drugs, we use a Chi square test to see if there is a difference between first choice use among psychiatrists and emergency physicians. Only 19 of the 71 psychiatrists (26.8%) classified benzodiazepines as their preferred product whereas 25 of the 37 emergency physicians (67.6%) did so. This difference in preference was significant (Chi square test p < 0.001).

No differences in preference between psychiatrists and emergency physicians was found for conventional antipsychotics (clothiapine, droperidol, haloperidol, zuclopenthixol en zuclopenthixol long acting) (psychiatrists; 27 (38%) and emergency physicians; 10 (27%); Chi square test p = 0.253) but a significant difference was found for atypical antipsychotics (aripiprazole, olanzapine, risperidone, quetiapine) (psychiatrists; 27 (38%) and emergency physicians; 7 (18.9%); Chi square test p = 0.042). The analysis of differences in preference for specific drugs in non-secluded patients are listed in Table 3. The first three columns use for the considered drug the criterium: did the participant rank this as number 1 drug? The last 3 columns use as criterium: did the participant put this drug in its top 3? The number of positive answers to this criterium together with the percentages of either professional group are given. A Fisher’s exact test was performed for each drug. Psychiatrists place quetiapine and zuclopenthixol (long acting formula) significantly more than emergency physicians in their top 3 of used drugs. In contrast, midazolam, diazepam and haloperidol are significantly more in the top 3 of emergency physicians.

**Table 3:** Between group differences in non-secluded patients

	Nr 1 favoured product			Top 3 favoured products		
	Psychiatrist (n = 71)	Emergency physician (n = 37)	Fisher’s exact p	Psychiatrist (n = 71)	Emergency physician (n = 37)	Fisher’s exact p
Alprazolam LA	0 (0)	0 (0)	–	1 (1.4)	0 (0)	–
Alprazolam	1 (1.4)	1 (2.7)	1.000	4 (5.6)	3 (8.1)	0.689
Aripiprazole	3 (4.2)	0 (0)	0.55	7 (9.9)	0 (0)	0.093
Bromazepam	0 (0)	0 (0)	–	1 (1.4)	0 (0)	–
Clonazepam	0 (0)	0 (0)	–	0 (0)	1 (2.7)	–
Clorazepaat	2 (2.8)	2 (5.4)	0.605	9 (12.7)	7 (18.9)	0.404
Clotiapine	17 (23.9)	4 (10.8)	0.128	28 (39.4)	15 (40.5)	1.000
Diazepam	0 (0)	5 (13.5)	0.004	3 (4.2)	17 (45.9)	<0.001
Droperidol	5 (7.0)	2 (5.4)	1.000	17 (23.9)	11 (29.7)	0.644
Escitalopram	0 (0)	0 (0)	–	0 (0)	2 (5.4)	0.115
Haloperidol	3 (4.2)	4 (10.8)	0.228	6 (8.5)	14 (37.8)	<0.001
Lorazepam	14 (19.7)	9 (24.3)	0.625	43 (60.6)	15 (40.5)	0.067
Midazolam	0 (0)	2 (5.4)	0.115	0 (0)	6 (16.2)	0.001
Olanzapine	17 (23.9)	7 (18.9)	0.631	40 (56.3)	15 (40.5)	0.156
Paliperidone	0 (0)	0 (0)	–	1 (1.4)	0 (0)	–
Pipamperone	0 (0)	0 (0)	–	3 (4.2)	1 (2.7)	1.000
Prazepam	0 (0)	0 (0)	–	1 (1.4)	0 (0)	–
Promethiazine	0 (0)	0 (0)	–	0 (0)	1 (2.7)	–
Quetiapine	5 (7.0)	0 (0)	0.163	22 (31)	0 (0)	<0.001
Risperidone	2 (2.8)	0 (0)	0.545	6 (8.5)	1 (2.7)	0.418
Trazodone	0 (0)	0 (0)	–	1 (1.4)	0 (0)	–
Sodium Valproate	0 (0)	1 (2.7)	–	4 (5.6)	1 (2.7)	0.659
Zuclopenthixol	0 (0)	0 (0)	–	2 (2.8)	1 (2.7)	1.000
Zuclopenthixol LA	2 (2.8)	0 (0)	0.545	14 (19.7)	0 (0)	0.002

Fisher’s exact test was performed when one of the two groups had at least two counts (two-sided).

In secluded patients, psychiatrists [n = 12 (16.9%)] classified a benzodiazepine as their preferred product whereas this was significantly higher in the emergency physicians group [n = 18 (48.6%)]; Chi square test p < 0.001).

A significant difference was found in preference between psychiatrists and emergency physicians for conventional antipsychotics (psychiatrists; 39 (54.9%) and emergency physicians; 11 (29.7%); Chi square test p = 0.013) but this was not the case for atypical antipsychotics (psychiatrists; 20 (28.2%) and emergency physicians; 7 (18.9%); Chi square test p = 0.292). The analysis of differences in preference for specific drugs in secluded patients are listed in Table 4 analogously to Table 3. Psychiatrists place olanzapine, zuclopenthixol (long acting formula) and quetiapine significantly more in their top 3 than emergency physicians. In contrast, diazepam, haloperidol and midazolam, are placed in the top 3 significantly more amongst emergency physicians.

**Table 4:** Between group differences in secluded patients

	Nr 1 favoured product			Top 3 favoured products		
	Psychiatrist (n = 71)	Emergency physician (n = 37)	Fisher’s exact p	Psychiatrist (n = 71)	Emergency physician (n = 37)	Fisher’s exact p
Amisulpride	0 (0)	0 (0)	–	1 (1.4)	0 (0)	–
Alprazolam	0 (0)	1 (2.7)	–	2 (2.8)	4 (10.8)	0.178
Aripiprazole	3 (4.2)	1 (2.7)	1.000	4 (5.6)	1 (2.7)	0.659
Bromazepam	0 (0)	0 (0)	–	1 (1.4)	0 (0)	–
Clonazepam	0 (0)	0 (0)	–	1 (1.4)	1 (2.7)	1.000
Cloxazolam	0 (0)	0 (0)	–	1 (1.4)	0 (0)	–
Clorazepaat	3 (4.2)	1 (2.7)	1.000	10 (14.1)	7 (18.9)	0.581
Clotiapine	16 (22.5)	7 (18.9)	0.806	31 (43.7)	16 (43.2)	1.000
Diazepam	0 (0)	6 (16.2)	0.001	6 (8.5)	20 (54.1)	<0.001
Droperidol	13 (18.3)	3 (8.1)	0.253	28 (39.4)	11 (29.7)	0.400
Escitalopram	0 (0)	0 (0)	–	0 (0)	0 (0)	–
Haloperidol	3 (4.2)	1 (2.7)	1.000	10 (14.1)	14 (37.8)	.007
Lamotrigine	0 (0)	0 (0)	–	0 (0)	1 (2.7)	–
Lorazepam	6 (8.5)	8 (21.6)	0.071	38 (53.5)	14 (37.8)	0.156
Midazolam	0 (0)	2 (5.4)	0.115	0 (0)	7 (18.9)	<0.001
Olanzapine	17 (23.9)	6 (16.2)	0.460	34 (47.9)	9 (24.3)	0.023
Prazepam	0 (0)	0 (0)	–	1 (1.4)	0 (0)	–
Promethiazine	0 (0)	0 (0)	–	1 (1.4)	3 (8.1)	0.115
Quetiapine	3 (4.2)	0 (0)	0.550	12 (16.9)	0 (0)	0.007
Risperidone	0 (0)	0 (0)	–	2 (2.8)	1 (2.7)	1.000
Zuclopenthixol	0 (0)	0 (0)	–	4 (5.6)	0 (0)	0.297
Zuclopenthixol LA	7 (9.9)	0 (0)	0.093	22 (31.0)	0 (0)	<0.001
Sodium Valproate	0	1 (2.7)	–	2 (2.8)	1 (2.7)	1.000
Paliperidone	0	0	–	1 (1.4)	0 (0)	–
Pipamperone	0	0	–	1 (1.4)	1 (2.7)	1.000

Fisher’s exact test was performed when one of the two groups had at least 2 counts (2-sided).

#### Differences in medication preferences for age-groups

The total sample of respondents was splitted into a group of prescribers that was younger than 45 years of age (n = 67, 62%) and a group older than 45 years of age (n = 41, 38.0%). When non-secluded patients were to be considered by the respondents, it was found that older physicians (n = 6) tended to prefer droperidol as their number 1 choice drug over their younger peers (n = 1, Fisher’s exact p = 0.012) whereas a trend was observed for lorazepam as number 1 choice drug in favour of the younger physicians (n = 18) versus older peers (n = 5, p = 0.091). Further, no significant age effect on prescription preferences was found for all other drugs. When secluded patients were to be considered by the respondents, no significant effect for doctor’s age was observed except for zuclopenthixol long formula in the top 3 of favourite drugs which was preferred by older peers (n = 15) versus younger physicians (n = 7, p = 0.002). The same trend as seen for lorazepam as number 1 choice drug in the non-secluded patients was noticed for secluded patients (p = 0.075, with n = 12 younger and n = 2 older physicians).

#### Which drug preferences are most likely to be affected by a seclusion?

In order to detect if a drug preference was significantly altered when considering seclusion status of the patient a McNemar test for binomial proportions for matched-pair data was performed. If we consider the drugs that were picked as number 1 and compare this preference between secluded and non-secluded patients then droperidol is more preferred in the secluded patients (p = 0.022) and lorazepam in the non-secluded patients (p = 0.022). If we consider preference as placing this drug in the top 3 we found that droperidol (p = 0.003) and zuclopenthixol long formula (p = 0.039) are preferred in secluded patients and olanzapine (p = 0.012) and quetiapine (p = 0.006) in non-secluded patients.

### Use of guidelines and monitoring for efficacy and safety

Guidelines are not frequently used. Only 29 respondents (26.9%) report the use of guidelines. Of these 29, 62.1% use local guidance or recommendations, 17.2% a recommendation issued by a national professional society or 20.7% a published guideline. The majority of respondents (97.2%) uses only clinical response evaluations to monitor the effect of the rapid tranquillisation. There is no use of physical monitoring.

## Discussion

Agitation is a regularly encountered clinical condition by psychiatrists as well as by emergency physicians since 64% of the respondents have up to ten cases per month in treatment and 21% up to twenty cases per month.

The results of this study show that there is no clear or systematic rationale for prescribing for acute agitation in Belgium. In this respect, variability in practice in Belgium is comparable to other countries [21]. This is the most important finding since considerable risks (use of physical restraint, cardiovascular severe side effects, higher likelihood of high dose prescribing and polypharmacy) are attached to prescribing in acute agitation without clear and evidence based rationale.

Antipsychotics are ranked most often first choice and benzodiazepines second choice when all respondents in the survey are considered. In non-secluded patients, preference is given to olanzapine, lorazepam and clotiapine. In secluded patients, who arguably demonstrate higher degrees of agitation compared to non-secluded patients, clotiapine, olanzapine and droperidol are prescribed the most. The preference of droperidol is of particular interest since this drug has been banned from use in North America and the UK because of concerns over its cardiotoxicity, more specifically the significant lengthening of the QTc interval in certain patients [22]. Again, this finding illustrates the lack of international evidence based rationale in guiding the treatment of agitation. A recent consensus statement of the psychopharmacology workgroup of the American Association for Emergency Psychiatry [23] recommended antipsychotics—and in particular olanzapine or risperidone—as first-line management of acute agitation. However, this consensus statement does not differentiate patients according to their level of agitation, as is the case in our study. On the basis of a non-systematical review of the literature, Bak et al. [24] also advise for olanzapine and lorazepam, although the authors recommend to use lorazepam only in non-psychotic agitation. In addition, in a review of the literature in the period 1960–2000 by Battaglia et al. [25], it was found that most evidence for a safe and effective treatment of acute agitation was found for haloperidol, olanzapine and lorazepam. This recommendation is also supported in the NICE guideline on acute agitation [26]. The findings from our study point out that clinicians have a complex relationship with these guidelines. When they are asked to consider non-secluded and therefore mildly agitated patients, prescribing preferences are in line with published evidence. In contrast with this, when asked to consider secluded patients with evidently higher levels of agitation, the compliance with guidelines disappears and potent sedative drugs as clotiapine and droperidol emerge as a preferred choice.

It should be noted that zuclopenthixol is not a drug of choice for agitated patients since it does not meet the requirements of a drug used in rapid tranquillisation (i.e. onset of action within 20–30 min, maximum plasma concentration within 2 h, short half-life). However, the long acting form of zuclopenthixol is found to be well-favored, both in non-secluded as in secluded patients. This is not in line with earlier mentioned recent recommendations found in the literature [23, 24, 27] where long acting drugs have no place in the acute treatment of agitation and thus there is a clear need for education. The observation that some medications (e.g. escitalopram) are used which are not considered suitable for managing acute agitation is also of importance in this light.

An interesting finding is that a comparison between type of medical specialist produced significant differences in prescription preferences. Most notably, emergency physicians prefer benzodiazepines most, both in secluded and in non-secluded patients. The analysis of these responses on the level of individual drugs shows that (1) no specific preference for benzodiazepines with shorter half life could be found (midazolam as well as diazepam are reported) and (2) that specifically psychiatrists prefer antipsychotics as quetiapine (in all patients), olanzapine (in patients with higher levels of agitation) and long acting zuclopenthixol the most. This gives evidence to support the hypothesis that both types of specialists use different strategies to treat acute agitation. To our knowledge, this distinction is never made in earlier prevalence studies although it seems relevant from our data to do so. It is known that psychiatrists and emergency physicians have a different education with respect to psychotropic medication or use these drugs in other indications which may lead to a bias in selection preference. Moreover, the content of the hospital formulary may differ between general hospitals and psychiatric hospitals, which could in turn also lead to the selection of different treatments. It can also be argued that the purpose of rapid tranquillisation for the psychiatrist might be to effectively sedate the patient but without hampering further diagnostic actions, whilst an emergency doctor is primarily focused on controlling the disruptive behaviour. Finally, physicians—in Belgium—are approached with different pharmaceutical information by different pharmaceutical companies. It can be postulated that this practice also has an effect on which drug is prescribed, certainly in the context of our finding that most respondents never use any published guideline.

The age of the prescribing physician also plays an important role. Older physicians prefer significantly more the use of the long acting form of zuclopenthixol when compared to their younger peers (in secluded patients). This could also be interpreted as an effect of education or changes in information strategies from pharmaceutical companies.

An explanation for our finding of a sparse use of monitoring—at least for assessment of efficacy of a treatment—can be found in a recent systematic review by Zeller and colleagues who reported a similar observation [28]. The authors hypothesize that, although agitation is a common behavioural emergency, there is a lack of easy-to-administer instruments that could improve treatment quality or predict treatment effects.

Our study has several limitations. The response rate is low, the definition of agitation that was used is not a clear clinical definition and there were no questions on how medication was delivered to the patient (per os, intramuscularly, intravenously).

## Conclusion

There is no clear or systematic rationale for prescribing for acute agitation in Belgium. Practice in treating acute agitation shows a complex relation with published evidence and guidelines. The level of agitation in patients and the type of physician prescribing the first pharmaceutical treatment both are clearly important variables and should be implemented in further research designs. A variety of causes can be put forward to explain this difference between both groups of physicians and this also warrants further research.

The high prevalence of the non-recommended use of zuclopenthixol acetate in the psychiatrist group raises concern and should be taken into account in future medical education. Moreover, it is of great concern that there is a substantial lack in the existence and in the use of assessment tools that measure the effect and safety of a treatment—preferably directly in the moment and in the patient and not post hoc by means of a measurement scale by a caregiver.

Note: Availibility of psychotropic drugs outside Belgium

Certain drugs mentioned in this study are bot available or in use in countries outside Belgium. Clotiapine is a dibenzothiazepine with ATC (Anatomical Therapeutic Chemical Classification) N05AH06, Bromazepam is a benzodiazepine with ATC (Anatomical Therapeutic Chemical Classification). N05BA08 and pipamperone is a bipiperidine with ATC (Anatomical Therapeutic Chemical Classification) N05AD05.
